# Pharmacokinetics of levosimendan in critically Ill children on extracorporeal membrane oxygenation: a prospective observational study

**DOI:** 10.3389/fped.2025.1542417

**Published:** 2025-07-30

**Authors:** Stéphane Bertin, David Haefliger, Maria-Helena Perez, Monia Guidi, Laurent A. Decosterd, Vivianne Chanez, Ermindo R. Di Paolo, Thierry Buclin, Françoise Livio

**Affiliations:** ^1^ Service of Clinical Pharmacology, Department of Medicine, Lausanne University Hospital and University of Lausanne, Lausanne, Switzerland; ^2^ Pediatric Intensive Care Unit, Department Woman-Mother-Child, Lausanne University Hospital and University of Lausanne, Lausanne, Switzerland; ^3^ Centre for Research and Innovation in Clinical Pharmaceutical Sciences, Lausanne University Hospital and University of Lausanne, Lausanne, Switzerland; ^4^ Service of Clinical Pharmacology, Department of Laboratory Medicine and Pathology, Lausanne University Hospital and University of Lausanne, Lausanne, Switzerland; ^5^ Pharmacy Service, Lausanne University Hospital and University of Lausanne, Lausanne, Switzerland

**Keywords:** levosimendan, extracorporeal membrane oxygenation, pediatrics, infant, pharmacokinetics

## Abstract

**Background:**

Levosimendan is used off-label in pediatrics and pharmacokinetic (PK) data in this population are scarce. The only study in critically ill patients on extracorporeal membrane oxygenation (ECMO) showed altered PK parameters. Our study aimed to characterize the PK profile of levosimendan and its metabolites in critically ill children on ECMO and assess the adequacy of current dosing practices.

**Methods:**

We conducted a prospective observational PK study in a Swiss tertiary pediatric intensive care unit. Children on ECMO for hemodynamic failure receiving continuous levosimendan infusion (0.1 µg/kg/min for 48 h) were included.

**Results:**

Five full-term newborns and one infant were included (median age 24 days). A total of seven sets of eight blood samples were collected. Median (range) steady-state levosimendan concentration was 16.68 (7.92–18.88) ng/ml. Non-compartmental analysis revealed a median clearance (CL) of 5.99 (5.18–11.32) ml/min/kg, volume of distribution (Vd) of 1.48 (1.07–6.27) L/kg, and elimination half-life of 4.59 (3.40–6.50) hours. ECMO and body composition of our patients, mostly post-cardiac surgery neonates, might explain the increased CL and Vd in our population compared to older children not on ECMO (CL 3.60 ml/min/kg, Vd 0.35 L/kg). Active metabolite OR-1896 concentrations were markedly lower than in neonates not on ECMO.

**Conclusions:**

In our cohort of critically ill children on ECMO, augmented CL and Vd were associated with low levosimendan concentrations. This suggests that dosage should be increased in this context, to achieve optimal exposure of levosimendan and its active metabolite.

## 1 Introduction

Levosimendan is an inodilator which enhances cardiac contractility by sensitizing calcium through binding to cardiac troponin C. It also reduces systemic and pulmonary vascular resistance by opening ATP-dependent potassium channels in vascular smooth muscle cells (1–3). Levosimendan is primarily metabolized in the liver into inactive metabolites (glutathione pathway). Approximately 5% of the dose is reduced by gut microbiota into OR-1855, an inactive metabolite with a long half-life (∼75 h). OR-1855 is further re-absorbed and metabolized through N-acetyltransferase 2 (NAT-2) into OR-1896, an active metabolite with a longer half-life (80 h) than levosimendan (1 h). Thus, OR-1896 extends the hemodynamic effects of levosimendan over at least one week and plays a major role in its therapeutic efficacy (4, 5). Levosimendan's unique pharmacokinetic (PK) and pharmacodynamic (PD) profiles make it a valuable option for managing heart failure, showing dose-dependent benefits in cardiac output and vascular resistance (1, 6). Levosimendan is used for acute heart failure and advanced chronic heart failure but remains off-label in pediatrics due to limited evidence. It is typically administered as a single continuous infusion over 24–48 h, at rates ranging from 0.05 to 0.20 µg/kg/min (7, 8).

Extracorporeal membrane oxygenation (ECMO) is a temporary life-support for critically ill patients with severe cardiac/respiratory failure. In children and neonates, it is frequently used postoperatively of congenital heart surgeries, with pediatric ECMO cases having roughly doubled over the last decade (9, 10).

Critical illness causes pathophysiological changes like organ dysfunction, abnormal fluid status, and altered plasma protein levels, leading to significant PK changes, particularly in drug volume of distribution (Vd) and clearance (CL). ECMO circuits can further affect these parameters, especially for lipophilic and highly protein-bound drugs such as levosimendan, which tend to be more sequestered in the circuit, leading to potential underexposure and therapeutic failure (11, 12). Ensuring effective levosimendan concentrations is relevant as this drug is often used to facilitate weaning from ECMO (13). The risk of ECMO weaning failure was reduced by 80% in 118 children who received levosimendan in an observational study (14).

To date, available levosimendan PK data in children on ECMO are limited to one single study, which showed that ECMO increased the elimination rate of levosimendan by 78%, suggesting significant sequestration in ECMO circuits (15). Given the scarcity of data, our study aimed to better characterize the PK profile of levosimendan and its metabolites in children on ECMO and assess whether levosimendan dosing regimens currently in use are adequate.

## 2 Methods

### 2.1 Study design and patients

This was a single-center prospective observational PK study in critically ill children on ECMO conducted at the Pediatric Intensive Care Unit (PICU) of Lausanne University Hospital in Switzerland. Eligible patients were children (0–18 years) treated with ECMO for severe cardiac failure receiving levosimendan as part of their clinical management. Levosimendan was administered as a continuous infusion over 48 h at a rate of 0.1 µg/kg/min. Inclusion period spanned from January 2023 to December 2023. Exclusion criteria were absence of informed consent.

The study protocol was approved by the local Ethics Committee (Commission cantonale d'Éthique de la Recherche du Canton de Vaud, CER-VD, study Nr. 2022-01262). The study was conducted according to the Declaration of Helsinki, principles of Good Clinical Practice, Swiss Human Research Act (HRA) and Human research Ordinance (HRO), as well as other locally applicable regulations. Written informed consent was obtained from legal representatives before inclusion, or with retroactive effect.

### 2.2 Data collection

A total of eight blood samples were collected in each patient, at t1, t2, t4, t24, t48, t49, t52, t72 h after starting levosimendan infusion. If a blood sample was not collected at the exact scheduled times, the actual collection time was recorded. A clinical pharmacologist was present during the blood sample collection process. Blood samples (1.2 ml in patients older than three months, and 0.6 ml in patients up to 3 months) were drawn from an arterial line, collected in EDTA-K tubes and kept on ice prior to centrifugation at 2,000 *g* for 5 min at +4°C. Plasma was then transferred into 1-ml polypropylene test tubes and immediately frozen at −80°C, in most cases within 30 min after collection, until analysis. Levosimendan and its metabolites OR-1855 and OR-1896 were simultaneously measured in plasma samples by ultra-high-performance liquid-chromatography coupled to tandem mass spectrometry (UHPLC-MS/MS), using a highly sensitive method developed in-house and fully validated on a concentration range of 0.1–100 ng/ml, with a lower limit of quantification of 0.1 ng/ml for each analyte (16). Measurements were of excellent performances in terms of trueness (94.3%–105.3%), repeatability (1.9%–7.2%) and intermediate fidelity (2.3%–9.7%). Clinical data related to patient demographics, weight and height, gestational age at birth, current diagnoses, levosimendan dosage and co-medications, laboratory results, pediatric index mortality (PIM) score, cardiac function, ECMO and, if applicable, renal replacement therapy (RRT) settings were recorded. All transfusions (red blood cell concentrates, platelet concentrates, albumin, fibrinogen, fresh frozen plasma) during the sampling period were recorded.

### 2.3 PK analysis

A descriptive non-compartmental PK analysis using standard equations was performed. Last blood samples were drawn 24 h after the end of infusions and due to the long half-life of levosimendan metabolites (∼80 h), PK analysis was restricted to levosimendan. Steady-state concentration of levosimendan was determined as mean of the concentrations measured at t4, t24, and t48. Last four measured concentrations (t48, t49, t52, t72) were used to calculate terminal elimination rate constant (*λ*_z_), and thus terminal elimination half-life of levosimendan $\left(T_{1/2}\;=\;\mathrm{Ln}(2\right)/\lambda_{z})$. Total area under the curve (AUC_tot_) and area under the moment curve (AUMC_tot_) were calculated using the linear trapezoidal rule with extrapolation to infinity. Total clearance (CL_tot_) of levosimendan was obtained by dividing the total dose administered by $\mathrm{AU}C_{\mathrm{tot}}(CL_{\mathrm{tot}}\;=\;D/\mathrm{AU}C_{\mathrm{tot}})$. Mean residence time (MRT) was calculated by dividing AUMC_tot_ by AUC_tot_ and subtracting half of the total infusion time $\left(\mathrm{MRT}\;=\;\mathrm{AUM}C_{\mathrm{tot}}/\mathrm{AUCtot}-t_{\inf}/2\right)$. Distribution volume at steady state (Vd_ss_) was obtained by multiplying MRT and CL_tot_ $\left(Vd_{\mathrm{ss}}\;=\;\mathrm{MRT}\;\times \;CL_{\mathrm{tot}}\right)$. Transfusions during the sampling period were summed to account for a so-called “transfusion clearance” (CL_Tx_), under the assumption that infused volumes approximately compensated circulating volume loss.

## 3 Results

### 3.1 Patients

Of a total of six included patients, one received levosimendan twice with an interval of 5 days between 1st and 2nd infusions. Thus, a total of seven sets of samples were measured. Table 1 reports demographic and clinical data. There were five full-term neonates and one older infant. Median age was 24 days (range 13–164 days). Median body weight and height were 3.4 kg (2.7–5.8 kg) and 51.8 cm (49.0–57.0 cm), respectively. Median body surface area (BSA) was 0.22 m^2^ (0.19–0.30 m^2^) based on Mosteller formula. One patient was on RRT, while median creatinine level was 41 µmol/L (22–146 µmol/L) for the other five patients. Median albumin level was 30 g/L (24–40 g/L). Although liver function was not formally assessed, ALT and bilirubin values were within the normal range, except for one patient. During the sampling period, patients required transfusion support ranging from 145 to 438 ml/kg (median 184 ml/kg). Mortality risk predicted with PIM III score is given in Table 1. Issue was lethal for five out of the six patients. The patients' clinical history is detailed in the supplementary material (Supplementary Data S1).

**Table 1:** Demographic and clinical data.

Patient	1	2	3	4	5	6
Demographics						
Sex	Boy	Girl	Girl	Boy	Girl	Girl
Age (days)	164	20	13	41	29	15
Weight (kg)	5.8	3.2	2.7	3.1	3.6	3.7
Height (cm)	57	53	49	52	51	52
BSA (m^2^)	0.30	0.22	0.19	0.21	0.23	0.23
Clinical data						
Intervention	Cardiac surgery	Cardiac catheterization	Cardiac surgery	Cardiac surgery	No	Cardiac surgery
Creatinine (µmol/L)	22	41	21	35	41	146
RRT	No	No	Yes	No	No	No
ALT (U/L)	10	76	13	10	22	10
Bilirubin (µmol/L)	15	38	12	8	6	na
Albumin (g/L)	33	26	24	24	34	40
Hemoglobin (g/L)	117	133	123	117	112	132
Transfusion (ml/kg)^a^	184; 145^b^	154	309	438	388	136
PIM III score (%)	0.9	1.5	3.8	3.2	81.8	2.0
ECMO						
ECMO mode	VA	VA	VA	VA	VA	VA
Oxygenator	Quadrox-ID pediatric	Quadrox-ID pediatric	Euroset newborn	Euroset newborn	Euroset newborn	Euroset newborn
Priming volume (ml)	281	281	290	290	290	290
ECMO indication	Failure to wean from EC	Cardiac arrest	Cardiogenic shock	Cardiac arrest	Cardiac arrest	Cardiac arrest

^a^ Amount of transfusion during each sampling period: sum of red blood cell concentrates, platelet concentrates, albumin, fibrinogen, and fresh frozen plasma (expressed in ml per kg of bodyweight)

^b^ Volume of transfusion during the 1st and 2nd administrations of levosimendan respectively. BSA, body surface area; RRT, renal replacement therapy; PIM III, pediatric index of mortality 3 score; ECMO, extracorporeal membrane oxygenation; VA, veno-arterial; EC, extracorporeal circulation; na, not available.

### 3.2 Concentrations of levosimendan and metabolites

**Figure 1:**
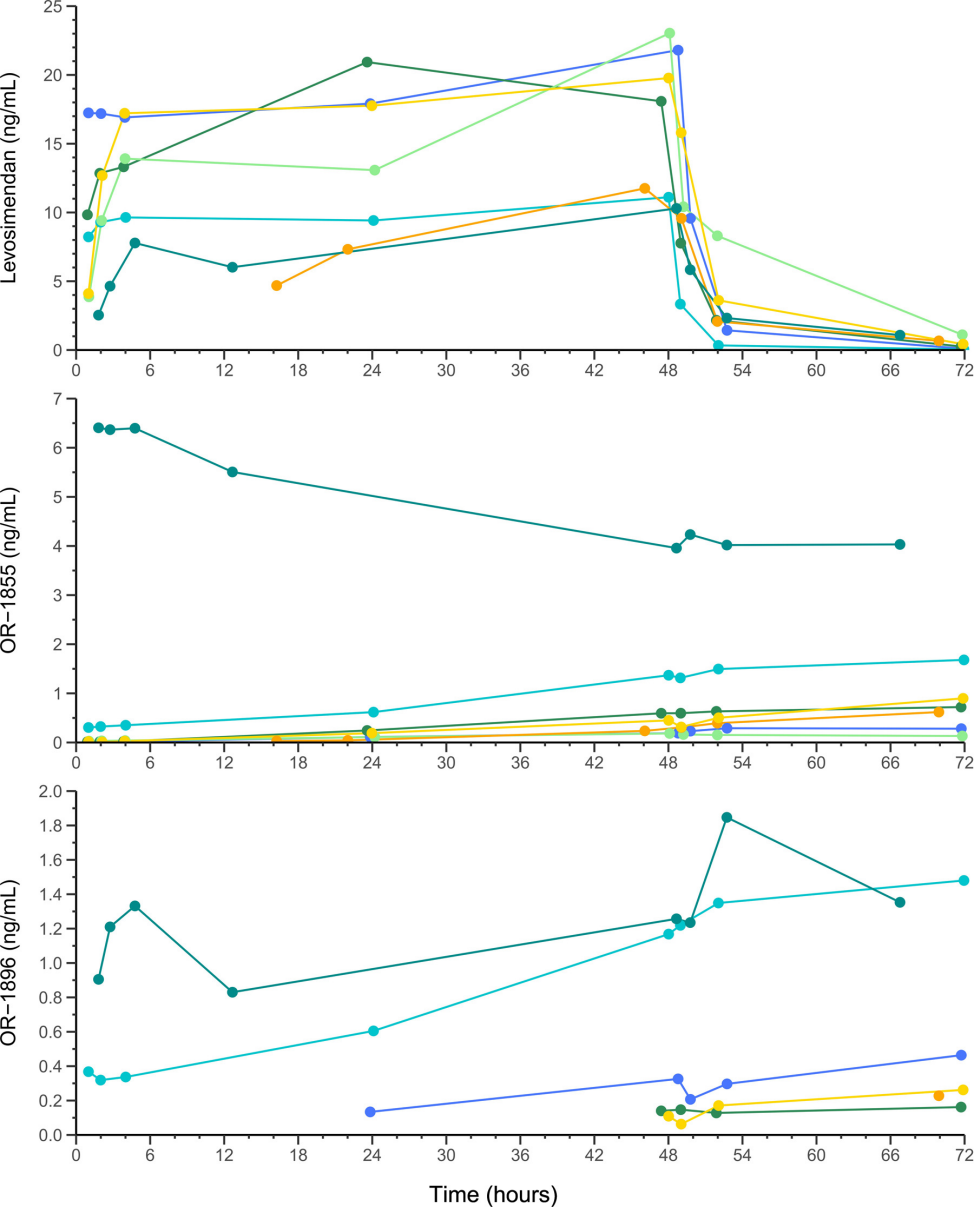
Concentration-time curves of levosimendan and metabolites (OR-1855, OR-1896) for all six patients (seven sets of samples). The different points correspond to the actual times of sample collections. Each patient is represented by a curve in a distinct color: the blue curve corresponds to Patient 1 (first sample), the light blue curve to Patient 1 (second sample), the dark green curve to Patient 2, the light green curve to Patient 3, the orange curve to Patient 4, the yellow curve to Patient 5, and the turquoise green curve to Patient 6. Of note, for Patient 6, metabolites were already detectable at time t0, as he had received an initial infusion of levosimendan 7 days prior to study inclusion.

A total of 54 samples were collected (6 samples instead of 8 in one patient). Among them, 29 measurements showed undetectable metabolites concentrations and 11 were under the limit of quantification of the method (mostly for OR-1855), especially in the first samples of each series. Figure 1 shows the concentration-time curves of levosimendan and its metabolites for all patients. Individual concentrations values are provided in the supplementary material (Supplementary Table S2). Median (range) steady-state concentration of levosimendan was 16.68 (7.92–18.88) ng/ml. Median (range) concentration of levosimendan at the end of infusion (t48) was 18.08 (11.11–23.04) ng/ml. Once the infusion was stopped, levosimendan decreased quickly and metabolites' levels were still increasing at the end of study period (Figure 1). Of note, OR-1896 concentrations could not be detected throughout the whole study period in one subject (Patient 3). Individual concentration-time curves of levosimendan and its metabolites are provided in the supplementary material (Supplementary Figures S3).

### 3.3 Levosimendan PK parameters and comparison with literature

Table 2 presents individual PK parameters. Median (range) CL_tot_ and CL_Tx_ were respectively 5.99 (5.18–11.32) ml/min/kg and 0.04 (0.03–0.10) ml/min/kg, giving a CL_Tx_/CL_tot_ ratio of 0.82% (0.28%–1.50%). Levosimendan median (range) half-life and Vdss were 4.59 (3.40–6.50) hours and 1.48 (1.07–6.27) L/kg, respectively. Table 3 presents the PK parameters of levosimendan from the present study and the literature. Removing the only patient on continuous RRT (Patient#3) did not substantially affect these estimates.

**Table 2:** Levosimendan individual calculated PK parameters and median values.

Patient	CL_tot_ (ml/min/kg)	CL_Tx_ (ml/min/kg)	CL_Tx_/CL_tot_ (%)	T_1/2_ (h)	Vd_ss_ (L/kg)
1	5.18	0.04	0.82	3.40	1.11
1 (run 2)	10.24	0.03	0.33	3.55	1.27
2	5.28	0.04	0.68	4.46	1.07
3	5.51	0.07	1.30	6.16	2.69
4	11.24	0.10	0.90	5.98	5.80
5	5.99	0.09	1.50	4.59	1.48
6	11.32	0.03	0.28	6.50	6.27
Median	5.99	0.04	0.82	4.59	1.48

CL_tot_, total clearance; CLTx, transfusion clearance; T_½,_ half-life; Vdss, steady-state volume of distribution.

**Table 3:** Studies reporting on pharmacokinetic parameters of levosimendan in pediatrics.

Study	Actual study	Bourgoin (15)	Bourgoin (15)	Turanlahti (25)	Turanlahti (25)	Wang (18)	Pellicer (19)
		(with ECMO)	(without ECMO)	(children)	(infants)		
Patients							
Number	6	14	7	6	7	94	11
Age (months)	0–5	1.5–63.5	1.5–63.5	13–78	3–6	2–11	<1
Setting	PICU^a^	PICU^b^	PICU^b^	Not PICU^c^	Not PICU^c^	PICU^d^	PICU^e^
ECMO	yes	yes	no	no	no	no	no
Dosis	0.1 µg/kg/min over 48 h	0.2 µg/kg/min over 24 h	0.2 µg/kg/min over 24 h	12 µg/kg over 10 min	12 µg/kg over 10 min	0.05 µg/kg/min over 48 h	0.2 µg/kg/min over 48 h
PK parameters							
CL_tot_ (ml/min/kg)	5.99	11.68^f^	5.72^f^	3.60	3.80	na	11.17
*λ*_z_ (h^−1^)	0.15	0.48	0.27	0.43^g^	0.30^g^	na	na
T_1/2_ (h)	4.59	1.44	2.60	1.60	2.30	16.47	na
Vd_ss_ (L/kg)	1.48	1.46^f^	1.27^f^	0.35	0.43	na	na

^a^ Patients in a PICU (mainly postoperative cardiac surgery or cardiac intervention).

^b^ Patients in a PICU (postoperative cardiac surgery or acute cardiac failure).

^c^ Patients not in a PICU (elective cardiac catheterization for the evaluation of cardiac surgery).

^d^ Patients in a PICU (postoperative cardiac surgery with cardio-pulmonary bypass).

^e^ Patients in a PICU (cardiac surgery with cardio-pulmonary bypass and stable preoperative hemodynamic condition).

^f^ Values calculated from population pharmacokinetic (PopPK) model of Bourgoin et al. (15).

^g^ Values calculated from available data $\left(\lambda_{z}\;=\;\ln(2)/T_{1/2}\right)$; na, not available. Reported studies are described in the supplementary material (Supplementary Data S4).

PK parameters (non-compartmental analysis) including total clearance of levosimendan (CL_tot_), elimination constant (λ_z_), terminal half-life (T_1/2_) and volume of distribution at steady-state (Vd_ss_). The study by Bourgoin et al. (15) included both patients who received ECMO support (*n* = 14) and those who did not (*n* = 7). In contrast, the study by Turanlahti et al. (25) included only patients not receiving ECMO, stratifying them into two age groups: 3–6 months (*n* = 7) and 13–78 months (*n* = 6).

## 4 Discussion

We investigated the pharmacokinetic profile of levosimendan and its metabolites in critically ill children, mainly neonates with congenital heart malformations who had just undergone surgery. All the patients were on ECMO and received a 48-h infusion of levosimendan at 0.1 µg/kg/min. Our descriptive non-compartmental analysis revealed a median steady-state concentration of 16.68 ng/ml, total CL of 5.99 ml/min/kg, Vd of 1.48 L/kg, and a half-life of 4.59 h.

Data from an *ex vivo* animal study showed that free concentrations of levosimendan and its active metabolite OR-1896 produced a positive cardiac inotropic effect, with half-maximal effective concentrations (EC_50_) of 4.20 ng/ml and 6.13 ng/ml, respectively (17). Since 98% of levosimendan and about 40% of OR-1896 are bound to albumin (4), free concentrations in our patient population are expected to be much lower than these EC_50_ values, even under conditions of reduced binding. Data on levosimendan plasma concentrations in children receiving continuous infusions are limited. Wang et al. observed maximal concentration (C_max_) of levosimendan at 14.94 ng/ml in 94 critically ill children (median age of 5 months) receiving 0.05 μg/kg/min over 48 h after congenital heart surgery (18). In 11 critically ill neonates (mean age of 15 days) undergoing surgical repair for congenital heart defects and receiving levosimendan with gradually increasing doses (0.1–0.2 µg/kg/min over 48 h), Pellicer et al. observed a C_max_ of levosimendan at 16.50 ng/ml (19). Differences in dosing regimens make it challenging to compare these data with ours. Since levosimendan PK is linear within the therapeutic dose range of 0.05–0.20 µg/kg/min (4), we could hypothesize our concentrations to be roughly half of Wang's and double of Pellicer's observations, when normalized to dosage. Indeed, considering that the dosage in Wang's study (0.05 µg/kg/min over 48 h) was half that of our study (0.1 µg/kg/min over 48 h), the C_max_ observed in Wang's study (14.94 ng/ml) would correspond about twice the exposure normalized to dosage in our study, where C_max_ was 18.08 ng/ml. Levosimendan sequestration by ECMO circuits could partly explain this difference, but other factors may also contribute (15). In Wang's study patients were scheduled for elective cardiac surgery, and levosimendan infusion was initiated postoperatively, starting upon PICU admission. Administered dose was 0.05 µg/kg/min over 48 h, but infusion rate varied at the discretion of the attending physician, e.g., based on adverse events. Consequently, some patients may have received doses lower than 0.05 µg/kg/min over some periods. Additionally, patients were older (median age of five months). All these factors could contribute to the difference in concentrations observed between Wang's data and ours. Differences in concentrations between Pellicer's study and ours are more difficult to explain. Both studies involved neonates with similar body weight. However, in Pellicer's study, patients were electively admitted for cardiac surgery with stable preoperative hemodynamics, whereas most of our patients experienced cardiac surgery or interventions complicated by hemodynamic instability. Additionally, in Pellicer's study, levosimendan infusion began before surgery at a dosis of 0.1 µg/kg/min. Only postoperatively, upon PICU admission, the dose was gradually increased (0.15 µg/kg/min, then 0.2 µg/kg/min). Thus, it cannot be concluded that Pellicer's patients actually received twice as much levosimendan as ours. In summary, variations in patient conditions, changes in levosimendan dosing over time, and ECMO use may all explain the observed discrepancies in levosimendan concentrations. Notably, our measured concentrations of levosimendan and its metabolites were significantly lower than the concentrations associated with hemodynamic effects in adult patients without ECMO (approximately 50–60 ng/ml for levosimendan and 6 ng/ml for OR-1896), indicating underdosing in our ECMO patients (20).

Our samples were collected up to 24 h after stopping levosimendan infusion. This was insufficient to capture steady-state concentrations of OR-1855 and OR-1896, as those metabolites have a long half-life (∼80 h). Consequently, metabolites’ concentration data were insufficient for a formal PK analysis. Nonetheless, metabolites appeared rather late and were low compared to Pellicer's study, where OR-1896 peaked at 3.58 ng/ml within 36 h (19). Sequestration by ECMO circuits possibly also affects levosimendan metabolites and partly explain our low observed concentrations (15). Levosimendan's high protein binding suggests limited dialyzability, consistent with similar concentrations observed between our single patient on continuous RRT and the other patients. However, this patient had a prolonged elimination half-life of levosimendan, which may reflect a larger volume of distribution, as reported in the literature (21). Renal function may also influence metabolites exposure. Only one patient was on RRT in our study and was notably the only one with no OR-1896 detected, suggesting a possible dialysis effect. PK data on levosimendan metabolites in RRT settings are limited, but some studies indicate they are dialyzable. Indeed, during a 4-h intermittent hemodialysis session, dialytic clearance of levosimendan metabolites was estimated to be 100 ml/min (21). This value exceeds the renal clearance of OR-1896 in healthy adults (15 ml/min) (22). These data and the moderate protein binding of OR-1896 suggest a likely removal of this metabolite during continuous RRT. Other factors, such as neonatal digestive tract immaturity and perioperative antibiotic use may also have impacted OR-1855 formation; while immature NAT-2 enzyme activity (not fully developed until around 4 years of age) and possibly its polymorphism might have contributed to the absence or low levels of OR-1896 (4, 23–25). As shown in the supplementary material (Supplementary Table S2), the metabolite ratio of OR-1896 over OR-1855 was fairly reproducible in each patient but the ratio differed between patients, in line with the known polymorphism on NAT-2. Since OR-1855 metabolite is formed by intestinal bacteria, digestive tract integrity and use of parenteral nutrition could have further impacted the gut flora and extent of levosimendan conversion to OR-1855. Altogether, these factors could explain our low and even undetectable metabolite concentrations.

Limited data exist on the PK of levosimendan in children, particularly those on ECMO. Available studies mentioned in Table 3 are described in the supplementary material (Supplementary Data S4). The only study on levosimendan PK in children on ECMO reported increased levosimendan CL, with an elimination constant rising from 0.27 h^−1^ (without ECMO) to 0.48 h^−1^ (with ECMO), indicating a 78% increase in elimination rate and a shortening of half-life from 2.60 to 1.44 h. In this study, effect of ECMO on levosimendan Vd was minimal with an increase from 1.27 L/kg (without ECMO) to 1.46 L/kg (with ECMO) (15). In addition, an *ex vivo* ECMO PK study led by the same group showed a levosimendan drug loss of 32% in a closed-loop circuit, in line with clinical PK data (unpublished data, “Sequestration of levosimendan and its metabolites, OR-1855 and OR-1896, on extracorporeal membrane oxygenation circuits: an *ex vivo* study”, abstract n° 286 presented on 18th of September 2024 at the Société Française d'Anesthésie et de Réanimation (SFAR) 2024 Congress). Besides ECMO circuits, additional factors unique to our neonatal population may have influenced levosimendan PK. As mentioned by Turanlahti et al., younger children (3–6 months) have a greater Vd and longer half-life compared to older children (13–78 months), suggesting influence of body composition (26). Indeed, neonates have the highest total body water percentage at birth (70%–80%), decreasing to adult levels (55%–60%) around 12 months (27). Although levosimendan is lipophilic, it is highly bound to albumin (97%–98%) and primarily distributed in body water (4). Increased Vd and half-life in our neonates may in part be due to their unique body composition. Additionally, increased levosimendan CL in our population (5.99 ml/min/kg) compared to Turanlahti's study (3.80 ml/min/kg) may be due to ECMO drug sequestration. However, this contrasts with Pellicer's findings, where neonates (without ECMO) showed higher levosimendan CL (11.17 ml/min/kg). Surprisingly, levosimendan CL in our population differed from the 11.68 ml/min/kg reported by Bourgoin et al. for children on ECMO and was closer to the 5.72 ml/min/kg estimate for non-ECMO children (15). Several factors might explain the discrepancies between our data and existing literature. First, differences in analytical methods could account for varying sensitivities across studies. Additionally, the patient populations differed in terms of age, weight, and BSA. Indeed, neonates seem to exhibit different PK parameters compared to older children, with higher CL and Vd. Moreover, while most patients were in PICU, the specific contexts varied among studies, such as post-operative cardiac surgery monitoring, elective cardiac catheterization, or PICU care for hemodynamic instability. These differences could contribute to further variability. For the effect of ECMO *per se*, levosimendan CL might be increased due to drug sequestration in the ECMO circuits. In neonates, lower blood flow rates on ECMO compared to adults could lead to clotting within the circuit, further adding a source of variability in drug sequestration. Our data also indicate that ECMO may increase levosimendan Vd, an effect that could be primarily explained by the high ratio of primed extracorporeal circuit volume (281–290 ml) to blood volume in neonates.

Patients on ECMO need effective anticoagulation to avoid circuit clotting, which increases bleeding risk. Postoperatively, transfusions are often required, possibly affecting levosimendan exposure due to hemodilution. Transfusion volumes ranged from 145 to 438 ml/kg in our study. Nevertheless, our estimated “transfusion clearance” (CL_Tx_) proved to be negligible compared to total levosimendan CL, averaging only 0.82% (range 0.28%–1.50%). Though based on rough assumptions, this estimate suggests that transfusions minimally affect levosimendan exposure.

There are several limitations in our study. First, given observed variability, this study did not include enough patients to fully explore levosimendan PK in children. In addition, there was no control (i.e., patients without ECMO), preventing the possibility to isolate specific effects of ECMO on levosimendan PK. Finally, the sampling period was too short to fully capture metabolite PK profiles.

In conclusion, we observed low concentrations of levosimendan and active metabolites, with increased levosimendan Vd and CL likely due to neonates' body composition and ECMO. Transfusions did not seem to impact levosimendan exposure. Higher doses and/or prolonged infusions are probably needed to ensure optimal therapeutic effect, to be confirmed by further data in this specific population. Additional studies are also needed to better characterize the exposure-response relationship of levosimendan in pediatric populations and to define potential target concentration ranges, particularly in neonates.

## Data Availability

The original contributions presented in the study are included in the article/Supplementary Material, further inquiries can be directed to the corresponding author.
