# Supplementary material for: Pharmacokinetics of levosimendan in critically Ill children on extracorporeal membrane oxygenation: a prospective observational study
Source: Front Pediatr. 2025 Jul 30;13:1542417. doi: 10.3389/fped.2025.1542417 (PMC12343485; doi:10.3389/fped.2025.1542417)
Supplement: Supplementary file 1 [file Datasheet1.pdf]

Supplementary Material

1 Supplementary Data S1

Detailed clinical history of the six included patients

Patient 1: an infant born at 32 + 5/7 weeks of gestational age and aged five and half months at study inclusion. He had several congenital malformations (unidentified polymalformative syndrome) including a ventricular septal defect (VSD) and congenital tracheal stenosis. He underwent slide tracheoplasty and VSD closure surgery needing a very long cardiopulmonary bypass (CPB) time and needed subsequently central veno-arterial (VA) extracorporeal membrane oxygenation (ECMO). Echocardiography showed heart failure with right systolic-diastolic dysfunction and tricuspid regurgitation, as well as left systolic dysfunction and mitral regurgitation. He received levosimendan twice at 5-day intervals. At the beginning of the 1st administration of levosimendan (samples t1, t2, and t4), an unquantified amount was accidentally infused outside the vein (para-venous leak). In addition, the infusion was suspended for one hour because of thoracic revision, with samples at t48, t49, and t52 delayed by 60 minutes. During the 2nd administration of levosimendan, sampling was conducted according to study protocol. The patient also experienced several episodes of bleeding requiring massive transfusions. During the 1st sampling period, he received 10 red blood cell concentrates, 1 platelet concentrate, and 12 fresh frozen plasma units, corresponding to a total volume of 1066 mL (184 mL/kg) over 72 hours. During the 2nd sampling period, he received 12 red blood cell concentrates, 3 platelet concentrates, and 4 fresh frozen plasma units, corresponding to a total volume of 843 mL (145 mL/kg) over 72 hours. Cardiac outcome allowed weaning off ECMO. However, outcome was fatal several weeks later due to infectious and cerebrovascular complications.

Patient 2: a term neonate (38 + 2/7 weeks of gestation) aged 20 days at study inclusion. She had a congenital heart malformation with pulmonary atresia and a large VSD. After cardiac catheterization, she presented a cardiopulmonary arrest (CPA), requiring cardiopulmonary resuscitation (CPR) and initiation of central VA ECMO. Post-CPA, biventricular dysfunction and left ventricular ejection fraction (LVEF) of 13% indicated a treatment with levosimendan. Administration and sampling of levosimendan were conducted according to protocol. Due to massive bleeding, the patient received multiple transfusions during the sampling period (5 red blood cell concentrates, 2 platelet concentrates, and 8 fresh frozen plasma units) corresponding to a total volume of 494 mL (154 mL/kg) over 72 hours. ECMO was rapidly weaned off, and outcome was favorable with an echocardiogram showing a close to normal biventricular function (LVEF of 55%) at discharge from pediatric intensive care unit (PICU).

Patient 3: a term neonate (40 + 4/7 weeks of gestation) aged 13 days at study inclusion, with a complex congenital heart defect. Open heart surgery was done at age 5 days with perioperative administration of levosimendan (without any sampling). Following CPB, acute renal failure due to refractory low cardiac output syndrome prompted ECMO with central VA canulation and renal replacement therapy (RRT). At age 9 days, redo surgery was complicated by hemorrhagic shock. Attempts to wean ECMO were unsuccessful, prompting administration of levosimendan at age 13 days (administration and sampling of levosimendan according to study protocol). During the sampling period, 8 red blood cell concentrates, 8 platelet concentrates, and 6 units of fresh frozen plasma were transfused, corresponding to a total volume of 847 mL (309 mL/kg) over 72 hours. Subsequent echocardiography after

levosimendan treatment showed persistent dysfunction. Despite all attempts and treatments, weaning off ECMO could not be achieved, and the child died after withdrawal of care.

Patient 4: a term newborn (39 + 1/7 weeks of gestation) aged 41 days at study inclusion. He was admitted to PICU for surgical management of a complex congenital heart disease with complete atrioventricular canal and hypoplastic left ventricle. A pre-operative echocardiography showed good cardiac function (LVEF 57%). During surgery (pulmonary artery banding and ductus arteriosus closure), the patient presented acute desaturation and CPA, prompting a central VA ECMO. Postoperatively, the patient was administered levosimendan. Administration was carried out according to study protocol, but only six samples could be collected. Successive echocardiographic evaluations initially showed very poor myocardial contractility, improving with levosimendan treatment. The patient needed massive transfusion during the sampling period (8 red blood cell concentrates, 7 platelet concentrates, and 14 units of fresh frozen plasma, corresponding to a total volume of 1359 mL (438 mL/kg) over 72 hours). The outcome was unfavorable with inability to wean off ECMO, hemorrhagic shock, and infectious complications leading to withdrawal of care and death 7 days after study inclusion.

Patient 5: a term neonate (40 + 3/7 weeks of gestation) aged 29 days at study inclusion. The patient was admitted to PICU at age 24 days with cardiogenic shock due to biventricular cardiac dysfunction in the context of a mitochondrial disorder. An echocardiography showed normal cardiac anatomy but severe biventricular dysfunction with a LVEF of 10%. The patient suffered cardiac arrest, necessitating CPR and central VA ECMO. Levosimendan was administered and sampled according to study protocol. During this period, the patient also presented hemorrhagic shock, requiring the transfusion of 11 red blood cell concentrates, 8 platelet concentrates, and 11 units of fresh frozen plasma, corresponding to a total volume of 1398 mL (388 mL/kg) over 72 hours. The patient's condition rapidly deteriorated, with inability to wean off ECMO along with multiple complications, including recurring severe hemorrhage and septic shock, leading to withdrawal of care 7 days after study inclusion.

Patient 6: a term neonate (39 + 3/7 weeks of gestation) aged 15 days at study inclusion. The patient was admitted to PICU for surgical treatment of a congenital heart defect (type 2 truncus arteriosus with stenosis of the bicuspid truncal valve, ventricular septal defect, and patent foramen ovale). During the perioperative period, there was a 1st administration of levosimendan (without any sample being collected). After surgery, the patient developed severe hypotension followed by cardiac arrest, attributed to a pulmonary hypertension crisis leading to secondary right heart failure. Unsuccessful CPR leaded to central VA ECMO, and levosimendan was administered for the 2nd time (i.e., 7 days after the 1st administration). Samples were collected following study protocol, although the t24 and t72 samples were taken a few hours earlier. During the sampling period, the patient required transfusion of 4 red blood cell concentrates, 2 platelet concentrates, and 8 units of fresh frozen plasma, corresponding to a total volume of 502 mL (136 mL/kg) over 72 hours. Patient's condition deteriorated, with inability to wean off ECMO, recurrent hemorrhages, anuria requiring RRT (which was initiated after levosimendan’s sampling), and infectious complications. She died 16 days after study inclusion.

2 Supplementary Data S2

Table S2: Individual concentrations of levosimendan and metabolites OR-1855 and OR-1896

Patient 1 (1st run)

	T1	T2	T4	T24	T48	T49	T52	T72
**Levosimendan [ng/mL]**	17.240	17.190	16.913	17.913	21.803	9.574	1.432	0.112
**OR-1855 [ng/mL]**	0.000	0.000	0.020*	0.112	0.190	0.231	0.290	0.281
**OR-1896 [ng/mL]**	0.000	0.000	0.000	0.134	0.326	0.207	0.297	0.464
**OR-1896 / OR-1855 [%]**	0	0	0	120	171	90	102	165

Patient 1 (2nd run)

	T1	T2	T4	T24	T48	T49	T52	T72
**Levosimendan [ng/mL]**	8.224	9.295	9.640	9.418	11.109	3.337	0.336	0.042*
**OR-1855 [ng/mL]**	0.305	0.324	0.351	0.618	1.369	1.315	1.495	1.681
**OR-1896 [ng/mL]**	0.368	0.319	0.337	0.605	1.168	1.219	1.349	1.480
**OR-1896 / OR-1855 [%]**	120	98	96	98	85	93	90	88

Patient 2

	T1	T2	T4	T24	T48	T49	T52	T72
**Levosimendan [ng/mL]**	9.833	12.850	13.318	20.933	18.082	7.768	2.164	0.255
**OR-1855 [ng/mL]**	0.000	0.016*	0.022*	0.244	0.595	0.596	0.633	0.721
**OR-1896 [ng/mL]**	0.000	0.000	0.000	0.000	0.140	0.147	0.128	0.162
**OR-1896 / OR-1855 [%]**	0	0	0	0	24	25	20	22

Patient 3

	T1	T2	T4	T24	T48	T49	T52	T72
**Levosimendan [ng/mL]**	3.870	9.413	13.913	13.079	23.037	10.416	8.302	1.111
**OR-1855 [ng/mL]**	0.028*	0.033*	0.039*	0.111	0.184	0.163	0.153	0.132
**OR-1896 [ng/mL]**	0.000	0.000	0.000	0.000	0.000	0.000	0.000	0.000
**OR-1896 / OR-1855 [%]**	0	0	0	0	0	0	0	0

Patient 4

	T1	T2	T4	T24	T48	T49	T52	T72
**Levosimendan [ng/mL]**	np**	np**	4.687	7.323	11.753	9.575	2.077	0.666
**OR-1855 [ng/mL]**	np**	np**	0.030*	0.043*	0.236	0.307	0.393	0.619
**OR-1896 [ng/mL]**	np**	np**	0.000	0.000	0.000	0.000	0.000	0.228
**OR-1896 / OR-1855 [%]**	na***	na***	0	0	0	0	0	37

Patient 5

	T1	T2	T4	T24	T48	T49	T52	T72
**Levosimendan [ng/mL]**	4.106	12.685	17.213	17.761	19.773	15.798	3.613	0.425
**OR-1855 [ng/mL]**	0.000	0.000	0.027*	0.190	0.450	0.315	0.503	0.898
**OR-1896 [ng/mL]**	0.000	0.000	0.000	0.000	0.109	0.063*	0.171	0.262
**OR-1896 / OR-1855 [%]**	0	0	0	0	24	20	34	29

Patient 6

	T1	T2	T4	T24	T48	T49	T52	T72
**Levosimendan [ng/mL]**	2.537	4.642	7.781	6.019	10.295	5.837	2.323	1.082
**OR-1855 [ng/mL]**	6.406	6.368	6.397	5.507	3.957	4.233	4.019	4.032
**OR-1896 [ng/mL]**	0.905	1.210	1.332	0.830	1.257	1.235	1.847	1.353
**OR-1896 / OR-1855 [%]**	14	19	21	15	32	29	46	34

Legend: * = observations below the lower limit of quantification (LLOQ) of the method (= 0.1 ng/mL). To be noted that lower limit of detection (LOD) was 0.05 ng/mL for levosimendan, 0.1 ng/mL for OR-1855, and 0.01 ng/mL for OR-1896. The only concentration measurement of levosimendan < LLOQ (t72 sample from Patient 1 on the 2nd administration) was included in the non-compartmental PK analysis (value close to the LOD). ** = not performed (np) and *** = not applicable (na)**.**

3 Supplementary Data S3

Figures S3: Individual concentration-time curves of levosimendan and its metabolites OR-1855 and OR-1896

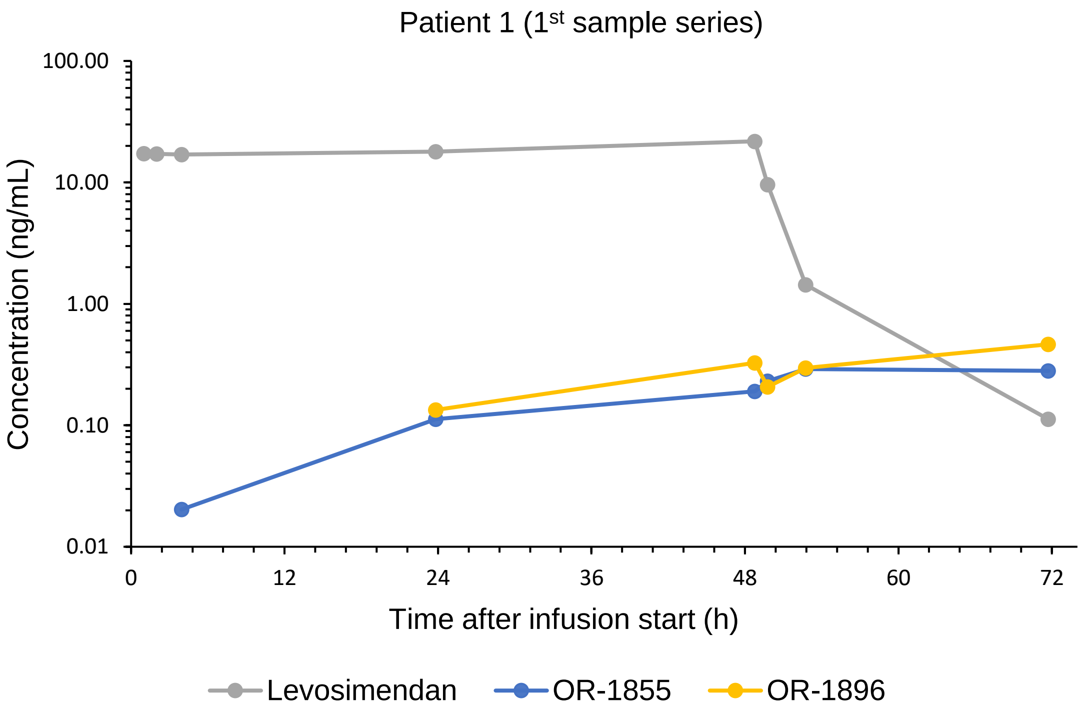

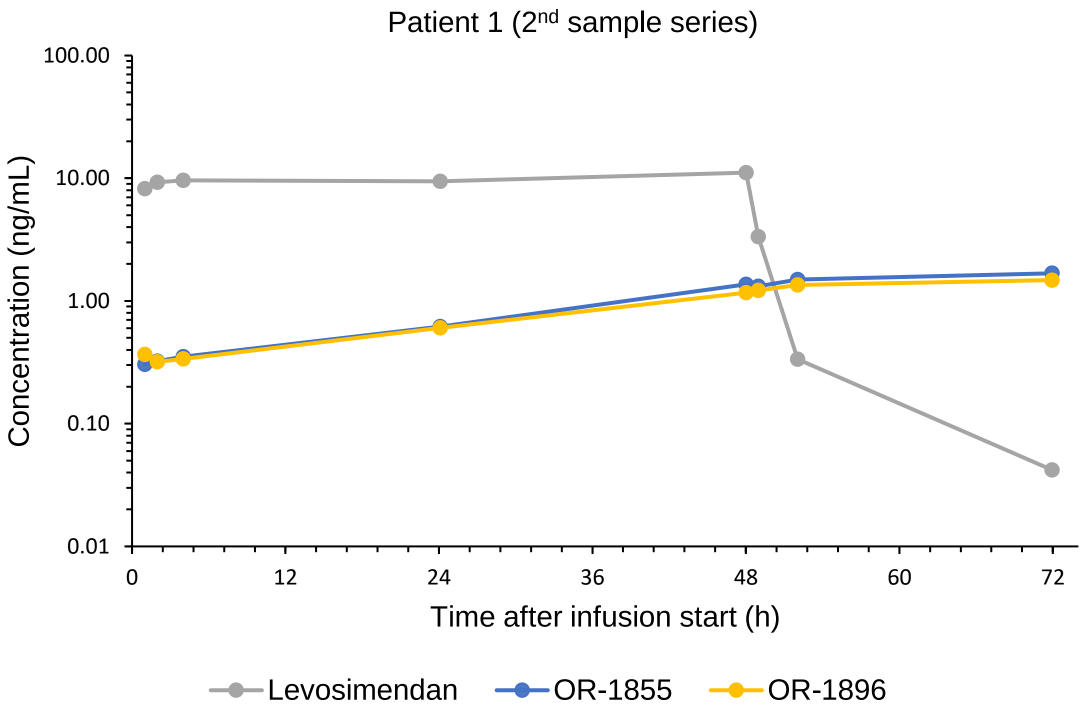
Patient 1 (2nd sample series)
100.00
10.00
1.00
0.10
0.01
Concentration (ng/mL)
0
12
24
36
48
60
72
Time after infusion start (h)
Levosimendan
OR-1855
OR-1896

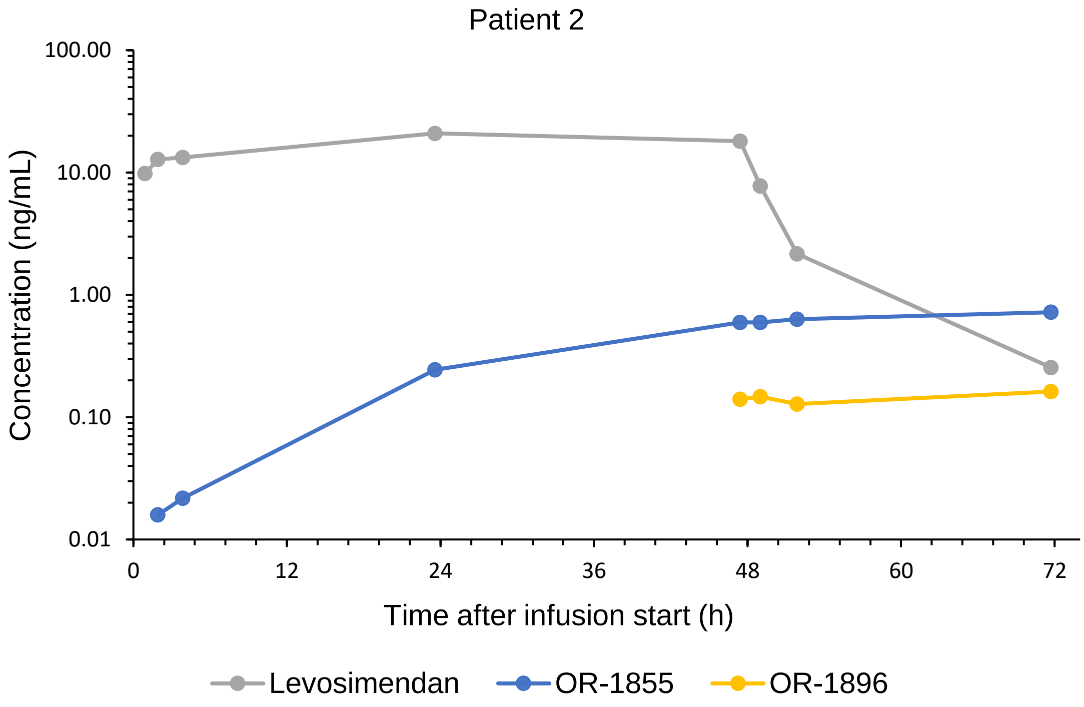
Patient 2
100.00
10.00
1.00
0.10
0.01
Concentration (ng/mL)
0
12
24
36
48
60
72
Time after infusion start (h)
Levosimendan
OR-1855
OR-1896

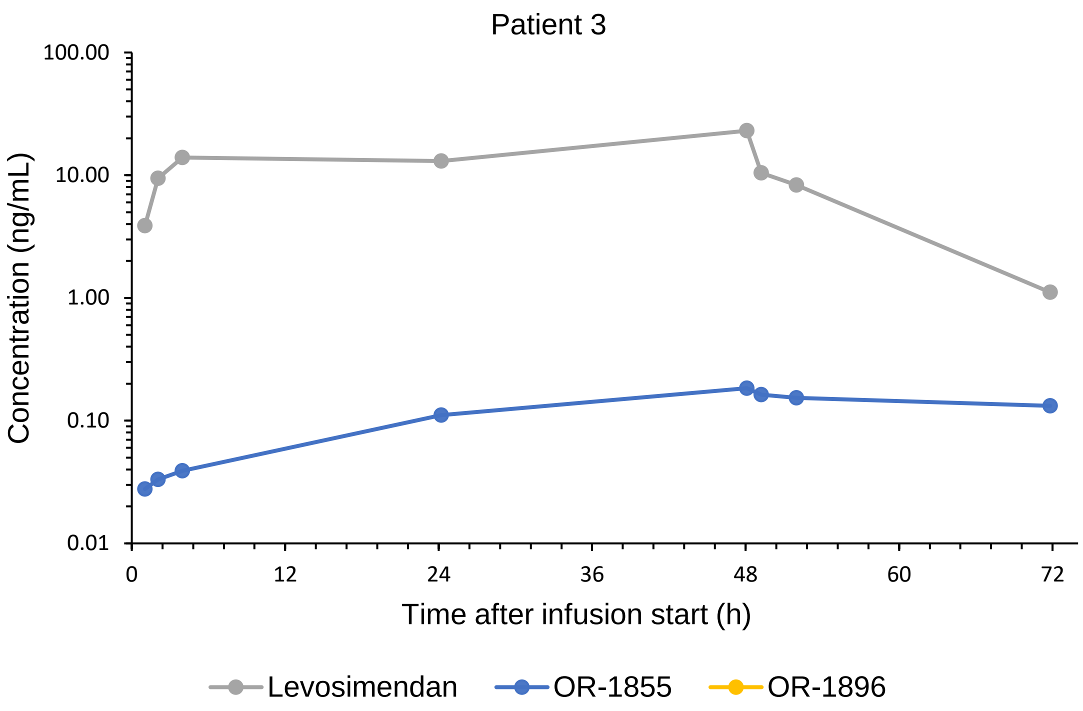
Patient 3
100.00
10.00
1.00
0.10
0.01
Concentration (ng/mL)
0
12
24
36
48
60
72
Time after infusion start (h)
Levosimendan
OR-1855
OR-1896

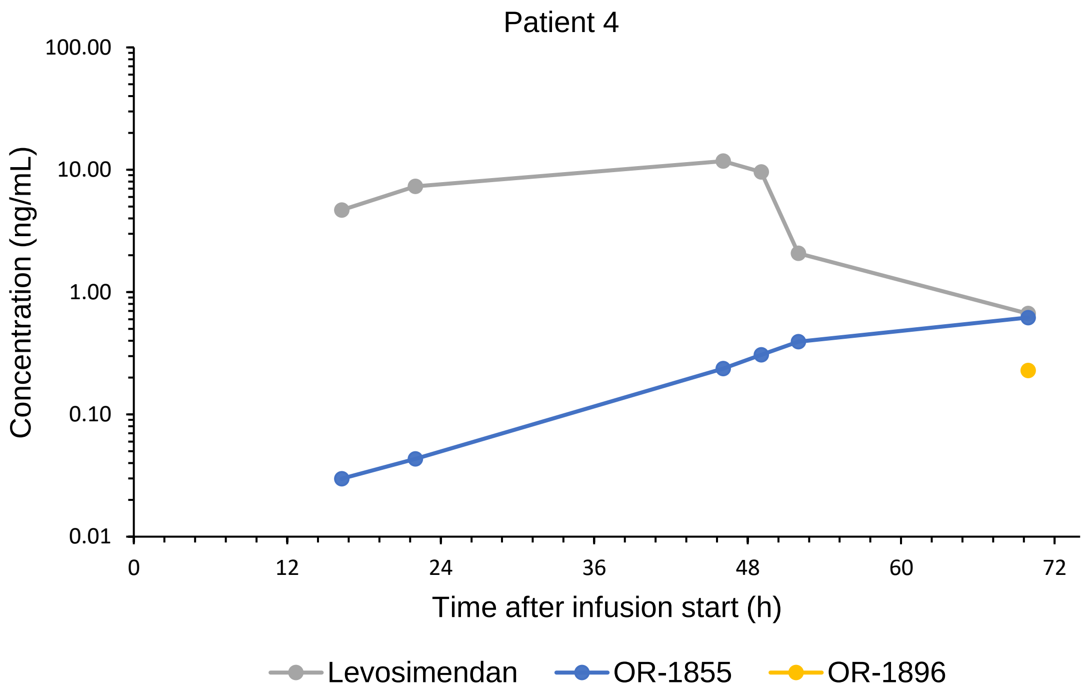
Patient 4
100.00
10.00
1.00
0.10
0.01
Concentration (ng/mL)
0
12
24
36
48
60
72
Time after infusion start (h)
Levosimendan
OR-1855
OR-1896

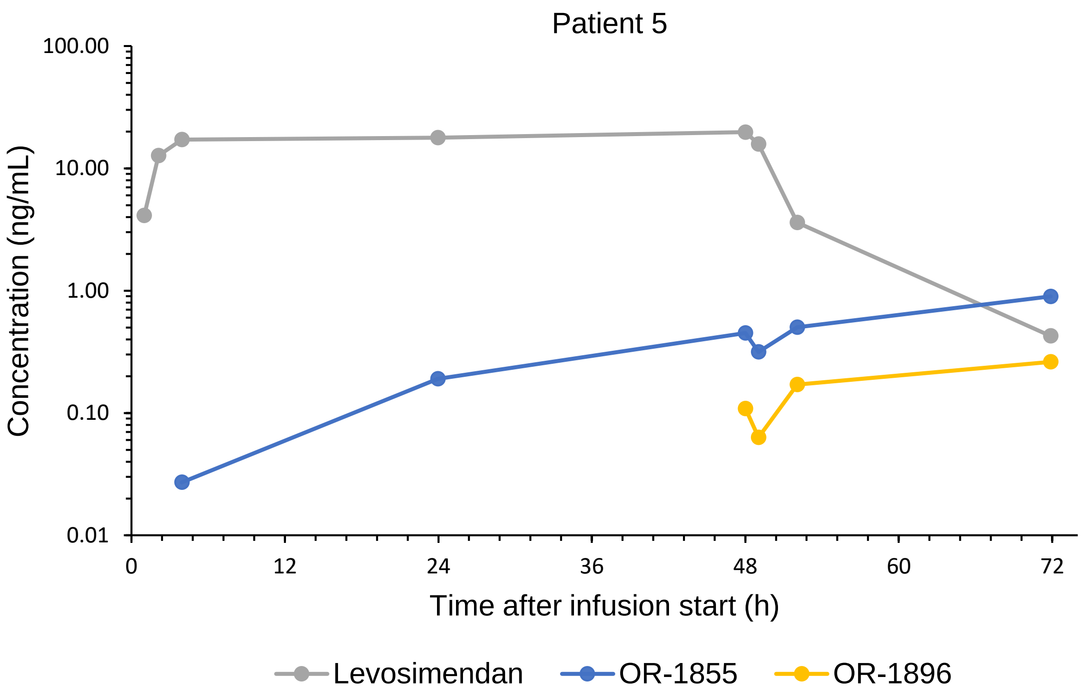
Patient 5
100.00
10.00
1.00
0.10
0.01
Concentration (ng/mL)
0
12
24
36
48
60
72
Time after infusion start (h)
Levosimendan
OR-1855
OR-1896

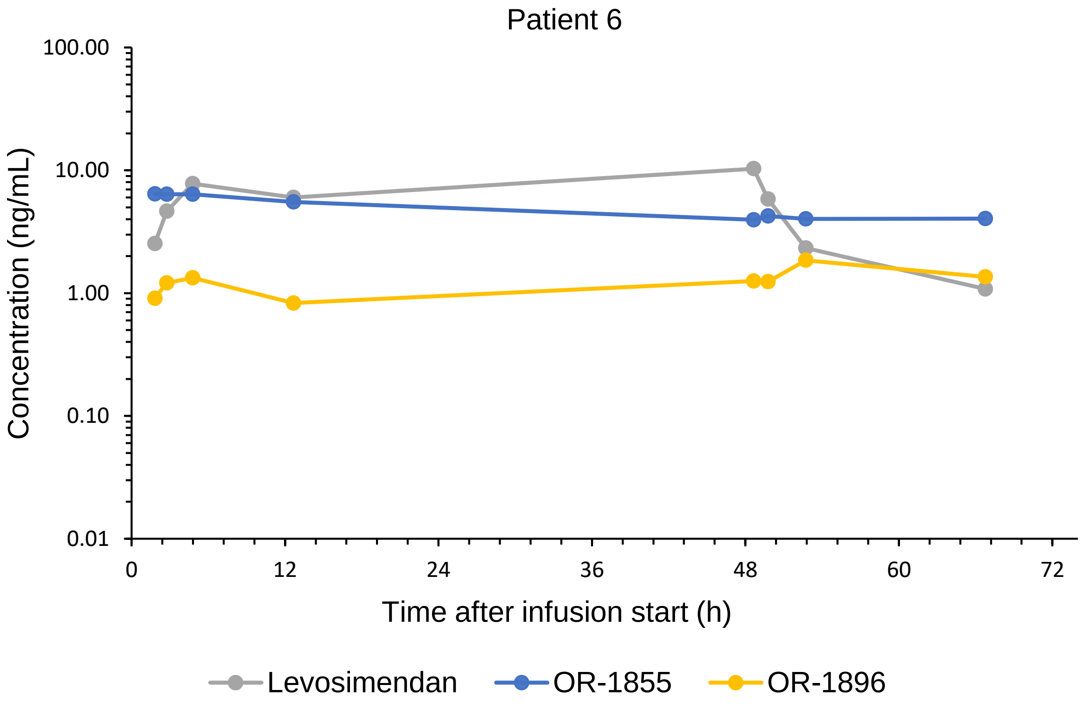
Patient 6
100.00
10.00
1.00
0.10
0.01
Concentration (ng/mL)
0
12
24
36
48
60
72
Time after infusion start (h)
Levosimendan
OR-1855
OR-1896

4 Supplementary Data S4

Summary of studies reporting on pharmacokinetic parameters of levosimendan in children

1) Turanlahti et al. *Pediatr Crit Care Med*. 2004:
A pharmacokinetic (PK) phase II study of levosimendan in pediatric subjects with congenital heart disease. The study enrolled 12 subjects (half with Down's syndrome) who were divided into two groups. The first one aged between 3-6 months (n = 5) and the second one aged between 6 months-7 years (n = 7). They were coming non-urgently to cardiac catheterization for the evaluation of cardiac surgery and were given a single intravenous dose of 12 µg/kg of levosimendan over 10 minutes. Mean terminal elimination half-life was 2.3 hours and 1.6 hours in the younger and older age groups, respectively. Total plasma clearance (CL) was similar between the two age groups (3.8 and 3.6 mL/min/kg) and steady-state volume of distribution (Vd) was 0.43 L/kg and 0.35 L/kg in the younger and older age groups, respectively.

2) Wang et al. *Crit Care*. 2019:
A study in which 94 patients received levosimendan as a continuous 0.05 µg/kg/min infusion over 48 hours following postoperative entry into PICU after congenital heart surgery (median age 5 months; cardiac surgery with cardiopulmonary bypass and median procedure duration of 80 minutes). The study was designed to assess the efficacy and safety of prophylactic administration of levosimendan to prevent low cardiac output syndrome after cardiac surgery. They also measured levosimendan and its metabolites up to 5 days after initiating levosimendan infusion. There was no formal PK analysis in this study (i.e., no report of CL and Vd). Nevertheless, they reported maximal levosimendan plasma concentration of 14.9 ng/mL and a mean terminal elimination half-life of 16.5 hours. This latter value should be considered with caution. As pointed out by the authors themselves, they did not observe any plateau concentrations, but a rapid drop in levosimendan plasma concentrations (not consistent with the very prolonged half-life they reported). They proposed several explanations for the absence of plateau and the rapid drop in concentrations, including variation of the infusion rate (which was at the discretion of the attending physician based on adverse events) and the impact of post-operative transfusion support. This latter conclusion is not concordant with our data showing minimal impact of "transfusion" clearance.

3) Pellicer et al. *Pediatr Res*. 2013:
A phase I pilot study with 11 neonates (mean age of 15 days) undergoing surgical repair for congenital heart defects with cardio-pulmonary bypass and receiving stepwise dose increases of levosimendan (0.1 to 0.2 µg/kg/min) as a continuous infusion over 48 hours. Patients were in stable preoperative hemodynamic condition and infusion was started intra-operatively at 0.1 µg/kg/min. The infusion was initiated immediately after the placement of central lines and continued for the entire duration of the surgical procedure, although the median duration of the surgery was not reported. The dosis was further increased to 0.15 µg/kg/min after surgery (upon PICU admission) and finally, 2 hours later, augmented to 0.2 µg/kg/min (for a total of 48 hours). One patient required ECMO for three days (individual PK data not provided). Levosimendan and its metabolites were measured daily up to 14 days after infusion start. Apparent CL of levosimendan (using non compartmental method) was 11.17 mL/min/kg. Vd value was not reported.

4) Bourgoin et al. *Clin Pharmacokinet*. 2023:

The only study reporting on PK of levosimendan in children with ECMO. It was a prospective multicentric population PK study including 21 patients receiving levosimendan at a rate of 0.2 µg/kg/min for 24 hours. Among them, 14 patients were supported by ECMO with a median age of 4.5 months, median weight of 6.1 kg and median body surface area of 0.42 m^2. Majority of patients were in a setting of post-operative cardiac surgery or acute cardiac failure (acute myocarditis, ischemic cardiomyopathy, sepsis). Their population PK analysis showed a two-compartment model with first-elimination order for levosimendan and a transit compartment (related to the digestive tract) to account for the delayed appearance of the metabolites. Of note, increasing weight was associated with a proportional increase in levosimendan Vd and CL, both in patients with and without ECMO. There was also an increase in levosimendan Vd and CL with ECMO, and an elimination constant rising from 0.27 h^{-1} (without ECMO) to 0.48 h^{-1} (with ECMO), which corresponded to a 78% increase in the elimination rate of levosimendan with ECMO. As a result, ECMO shortened the half-life of levosimendan from 2.60 to 1.44 hours. Based on their data and their PK model, we calculated a Vd of 1.27 L/kg and 1.46 L/kg in patients without and with ECMO, respectively. The value of total CL was 5.72 mL/min/kg and 11.68 mL/min/kg for patients without and with ECMO, respectively. Based on their PK model, the authors conducted simulations and found a 44% decrease in steady-state concentrations of levosimendan under ECMO. In view of this results, Bourgoin *et al*. suggested a high level of drug sequestration of levosimendan by ECMO circuits. To address the reduced levosimendan exposure caused by ECMO, they recommended to extend the infusion time to 48 hours while maintaining the same dosage (0.2 µg/kg/min).
